# C-MYC and BCL-2 mediate YAP-regulated tumorigenesis in OSCC

**DOI:** 10.18632/oncotarget.23089

**Published:** 2017-12-07

**Authors:** Xiyan Chen, Weiting Gu, Qi Wang, Xucheng Fu, Ying Wang, Xin Xu, Yong Wen

**Affiliations:** ^1^ School of Stomatology, Shandong University, Jinan, China; ^2^ Shandong Provincial Key Laboratory of Oral Tissue Regeneration, Jinan, China; ^3^ Qilu Hospital of Shandong University, Jinan, China

**Keywords:** yes-associated protein(YAP), oral squamous cell carcinoma (OSCC), C-MYC, BCL-2, tumorigenesis

## Abstract

Transcriptional co-activator Yes-associated protein (YAP) is a key oncogene in mammalian cells. The present understanding of YAP in oral squamous cells carcinoma (OSCC) remains unclear. The purpose of this study is to investigate the effects of YAP on proliferation and apoptosis in OSCC and the molecular mechanism. The results showed the expression level of YAP was higher in OSCC tissues than that in adjacent normal tissues. Knockdown of YAP in CAL27 cell lines prohibited cell proliferation while augmented apoptosis. Conversely, overexpression of YAP protected cells from apoptosis and promoted cell proliferation. Moreover, C-MYC and BCL-2 mRNA and protein levels were altered due to the differential expression of YAP. Subsequent Verteporfin treatment in CAL27 cells revealed that the transcription and translation of BCL-2 and C-MYC both decreased. In a tumor xenograft model, knockdown of YAP suppressed tumor growth of CAL27 *in vivo*, while YAP overexpression promoted the tumor growth. These results suggest that YAP is a crucial regulator that exerts pro-proliferation and anti-apoptosis effects in OSCC through actions affecting the cell cycle and intrinsic apoptotic signaling. Thus YAP could potentially serve as a valuable molecular biomarker or therapeutic target in the treatment of OSCC.

## INTRODUCTION

Oral squamous cell carcinoma is one of the main causes of cancer-related morbidity and mortality diseases worldwide. More than 250,000 new cases are diagnosed each year and, about 50% of patients died within 5 years [1]. Maximal tumor resection followed by radiotherapy and chemotherapy is the standard of care for OSCC [2]. Although great progress has been made in the treatment, the effect is not satisfactory. Further understanding of the pathogenesis of oral squamous cell carcinoma may help to predict cancer progression and provide new molecular targets for cancer therapy.

The Hippo signaling pathway is initially found as a major regulator in organ size control, but recent studies have found it is closely related to tumor progression [3, 4]. Yes-associated protein (YAP), which located in 11q22, is a major downstream effector of the Hippo signaling pathway. When the Hippo signaling pathway is inactivated, YAP can be transferred from the cytoplasm to the nucleus, activating gene expression by binding to many DNA-binding transcription factors, regulating diversified of cellular functions including proliferation, apoptosis, migration and differentiation [4, 5]. YAP is highly expressed in a variety of tumor tissues [6–8], and it has also been proved that YAP could regulate epithelial-mesenchymal transition (EMT) [9] and closely relate to the signal pathways associated with tumor development [10, 11]. Therefore, we decided to explore the possible role of YAP in OSCC.

Verteporfin is used clinically as a photodynamic therapy for the treatment of macular degeneration that eliminates the abnormal blood vessels. Recently, it has been found out that Verteporfin could abolish the liver hyperplasia induced by YAP overexpression [3]. These studies indicated that Verteporfin could inhibit the interaction of YAP-TEAD complex.

C-MYC, one of the most frequently inordinate oncogenes, is found highly expressed in OSCC tissues [12]. Elevated expression of C-MYC leads to the activation of checkpoints, which causes cell growth or cell death [13]. It has been shown that YAP can upregulate the expression of C-MYC in hepatocarcinoma cells and promote the development of tumor [14, 15].

In addition to dysregulation differentiation and aberrant proliferation, apoptosis is an important feature of cancer cells [16]. The balance of BCL-2 family proteins (such as proapoptotic proteins BAK and BAD), as well as anti-apoptotic proteins BCL-2 and MCL-17, regulates tumor cell apoptosis [17]. It has been reported that BCL-2 is highly expressed in more than 50% of human OSCC tissues [18]. The previous study has shown that BCL-2 is a crucial downstream target of the YAP-TEAD1 transcriptional active complex to regulate cell apoptosis in endometrial stromal cells [19].

In this research, the expression level of YAP in OSCC clinical tissues was detected and the effects of YAP on OSCC derived cell lines were investigated. The results stated clearly that YAP was more highly expressed in OSCC than in adjacent normal tissues. We also demonstrated that YAP could protect OSCC cells from apoptosis and promote cell proliferation. Moreover, the molecular mechanism of how ectopic YAP expression could lead to tumorigenesis was primarily explored.

## RESULTS

### Upregulation of YAP in human oral squamous cell carcinoma species

**Figure 1:**
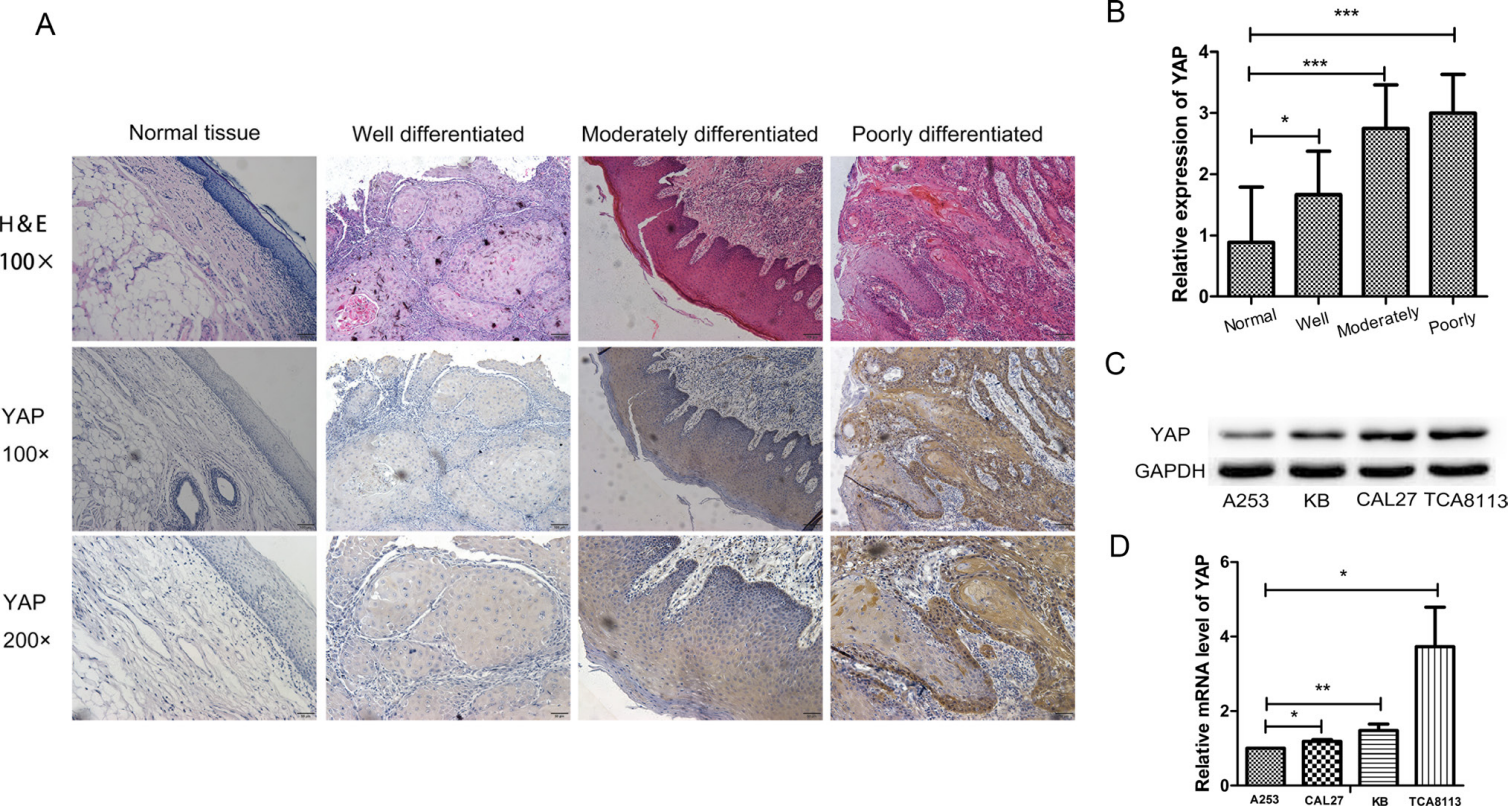
YAP was highly expressed in oral squamous cell carcinoma (**A**) Representative images of YAP immunostaining in normal tissues, well differentiated OSCC tissues, moderately differentiated OSCC tissues and poorly differentiated OSCC tissues. Low power (100×) scale bars 100 μm, high power (200×) scale bars: 50 μm. (**B**) Statistical relative quantification of YAP expression. (**C**) Western blot analysis of expression of YAP in four OSCC cell lines. (**D**) RT-PCR analysis of YAP expression in four OSCC cell lines. ^*^p < 0.05, ^**^P < 0.01, ^***^p < 0.001.

The expression level of YAP was investigated in three differentiated OSCC and one normal human tissues to determine the potential role of YAP in OSCC. Through immunohistochemistry (IHC) study, we found that YAP was significantly higher in the three differently differentiated OSCC tissues as compared with the normal tissues (Figure 1A and 1B). Moreover, YAP displayed a more nuclear-diffused staining in poorly differentiated OSCC tissues, while predominant cytoplasmic staining was seen in well differentiated OSCC tissues (Figure 1A). Quantitative real-time PCR (RT-PCR) and Western blot results revealed that YAP was highest in TCA8113 cell lines and lowest in A253 cell lines (Figure 1C and 1D).

### The expression level of YAP mRNA and protein in transfected CAL27 cell lines

**Figure 2:**
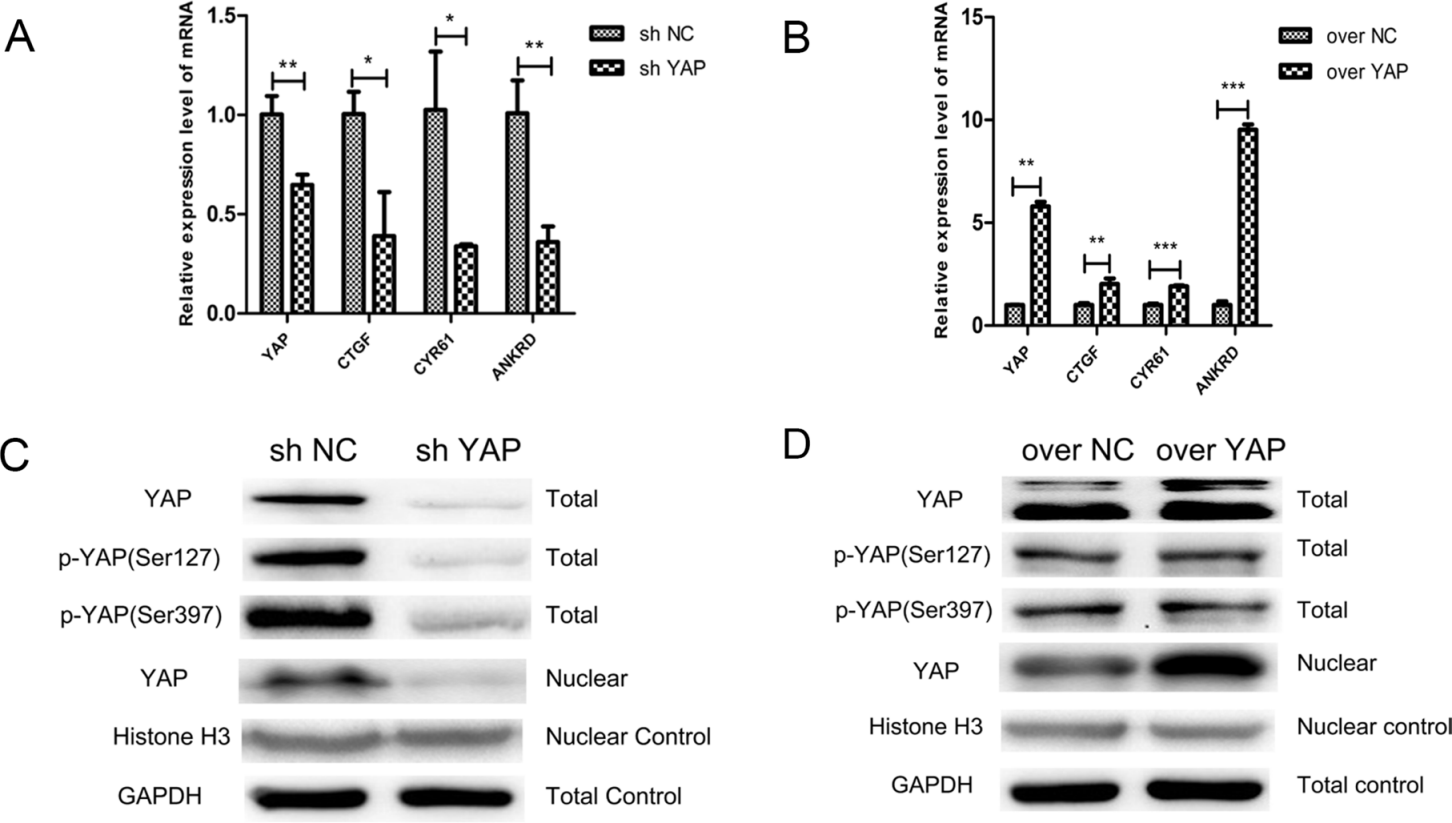
Knockdown and overexpression of YAP were generated in CAL27 cells (**A**, **B**) RT- PCR was conducted to determine the mRNA levels of YAP and the downstream genes of Hippo pathway in CAL27 cells. (**C**, **D**) CAL27 cells with overexpression or knockdown of YAP were used to analyze the expression of YAP, p-YAP (Ser127), p-YAP (Ser397). GAPDH was used as a total control and Histone H3 was used as a nuclear control. ^*^p < 0.05, ^**^P < 0.01, ^***^p < 0.001.

As YAP was found notably upregulation in human patients with OSCC, we aimed at discovering the effects of YAP on oral squamous cell lines. After mRNA (Figure 1C) and protein (Figure 1D) detection of YAP, CAL27 cell lines were chosen for further investigations. Stable cell populations of CAL27/YAP cells were generated after puromycin screening. The results of RT-PCR showed that the expression level of YAP and its target gene CTGF, CYR61, ANKRD in the YAP siRNA fragments transfected CAL27 cell lines (sh YAP) were lower than their counterpart siRNA transfected control cells (sh NC) (Figure 2A). And the LV5-YAP transfected CAL27 cell lines (over YAP) showed a higher YAP, CTGF, CYR61, ANKRD expression level than its counterpart empty vector transfected control cells (over NC) (Figure 2B). At the same time, knockdown of YAP (sh YAP) led to the decrease of YAP, phosphorylated YAP and YAP nuclear location (Figure 2C). In the contrast, overexpression of YAP led to the obvious upregulation of YAP and its nuclear location, but not phosphorylated YAP (Figure 2D). These results indicated that stable expression of YAP knockdown or overexpression cell lines were generated.

### Knockdown of YAP inhibited CAL27 cells proliferation

**Figure 3:**
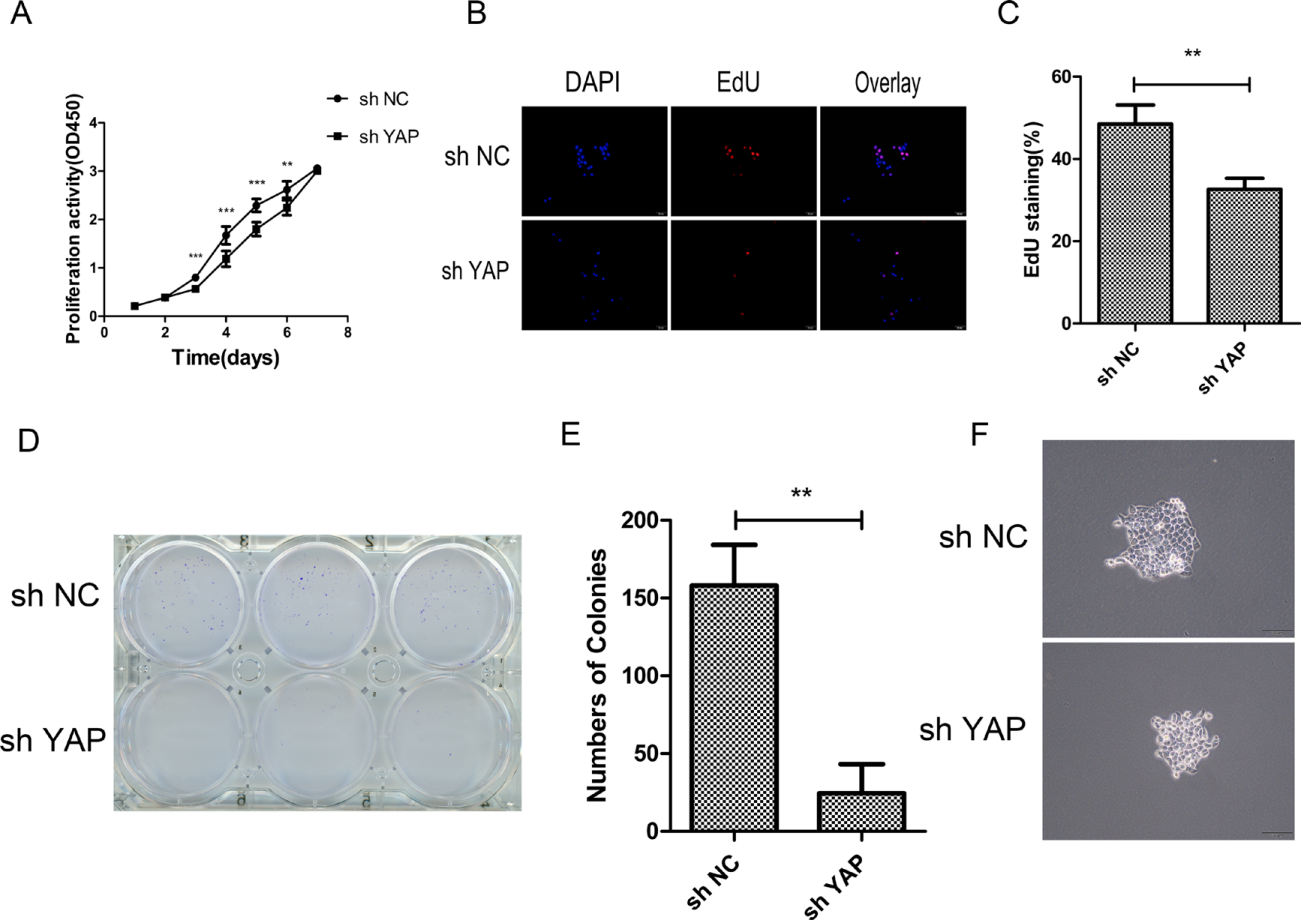
Knockdown of YAP inhibited cells proliferation (**A**) Cell proliferation was detected in CAL27 cells by CCK-8 assays. (**B**) The proliferating CAL27 cells were labeled with EdU. The images were representative of the results obtained. Scale bar: 50 μm. (**C**) Numbers of EdU-positive CAL27 cells. Shown is the proportion of EdU-positive CAL27 cells to all nuclei. (**D**) Colony formation assays of CAL27 cells. (**E**) Statically analysis of colony formation assay. The colonies consisting of more than 50 cells were counted. (**F**) Images of single colony of CAL27 cells. ^*^p < 0.05, ^**^P < 0.01, ^***^p < 0.001.

In order to further study the effects of YAP knockdown on CAL27cell lines, the CCK-8 assay, EdU staining and colony formation analyses were performed *in vitro*. CCK-8 assay revealed that knockdown of YAP significantly inhibited the cell proliferation activity at 3, 4, 5, and 6 days as compared with negative control group (Figure 3A). The result of EdU staining showed that the numbers of EdU-positive cells in sh YAP group was significantly less as compared with the control group (Figure 3B and 3C). Meanwhile, colony formation assay indicated that sh YAP group formed fewer colonies than those of the control group, and the single colony was smaller in sh YAP group than in the control (sh NC) group (Figure 3D, 3E and 3F).

### YAP overexpression promoted the proliferation of CAL27 cell lines

**Figure 4:**
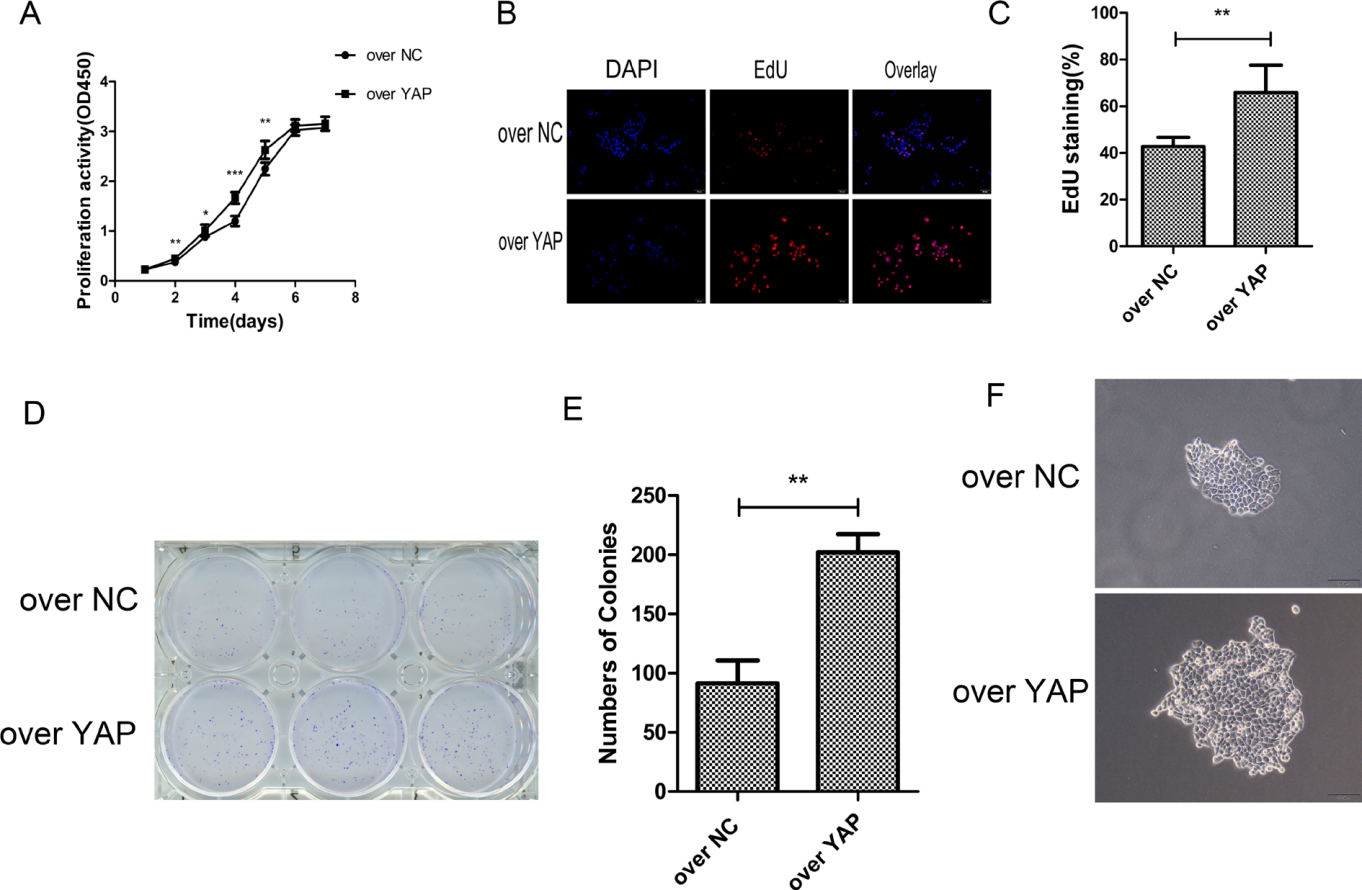
YAP overexpression promoted cells proliferation (**A**) The proliferation of CAL27 cells was measured by CCK-8 assay. (**B**) YAP overexpression resulted in increased CAL27 cells proliferation. Shown are representative images of the EdU staining. Scale bar: 50 μm. (**C**) Percent of EdU positive cells in CAL27 cells. (**D**) The effects of YAP upregulated on the colony formation of CAL27 cells. (**E**) Numbers of colonies. Data were listed as mean ± SD of three wells. (**F**) The effects of YAP overexpression on single colony. ^*^p < 0.05, ^**^P < 0.01, ^***^p < 0.001.

At the same time, the effect of YAP overexpression on cell proliferation was tested. Overexpression of YAP increased cell proliferation as compared to that of the control (over NC) group (Figure 4A). The effect of YAP overexpression on cell proliferation was confirmed by EdU staining assay. The result showed that EdU-positive cells in YAP overexpression group (over YAP) was more than those of the control group (over NC) (Figure 4B and 4C). Colony formation assay showed that there were more colonies formed in YAP overexpression group (ove YAP), and the single colony was bigger in over YAP than in the control (over NC) group (Figure 4D, 4E and 4F).

### YAP promoted cell cycle transition and inhibited cells apoptosis

**Figure 5:**
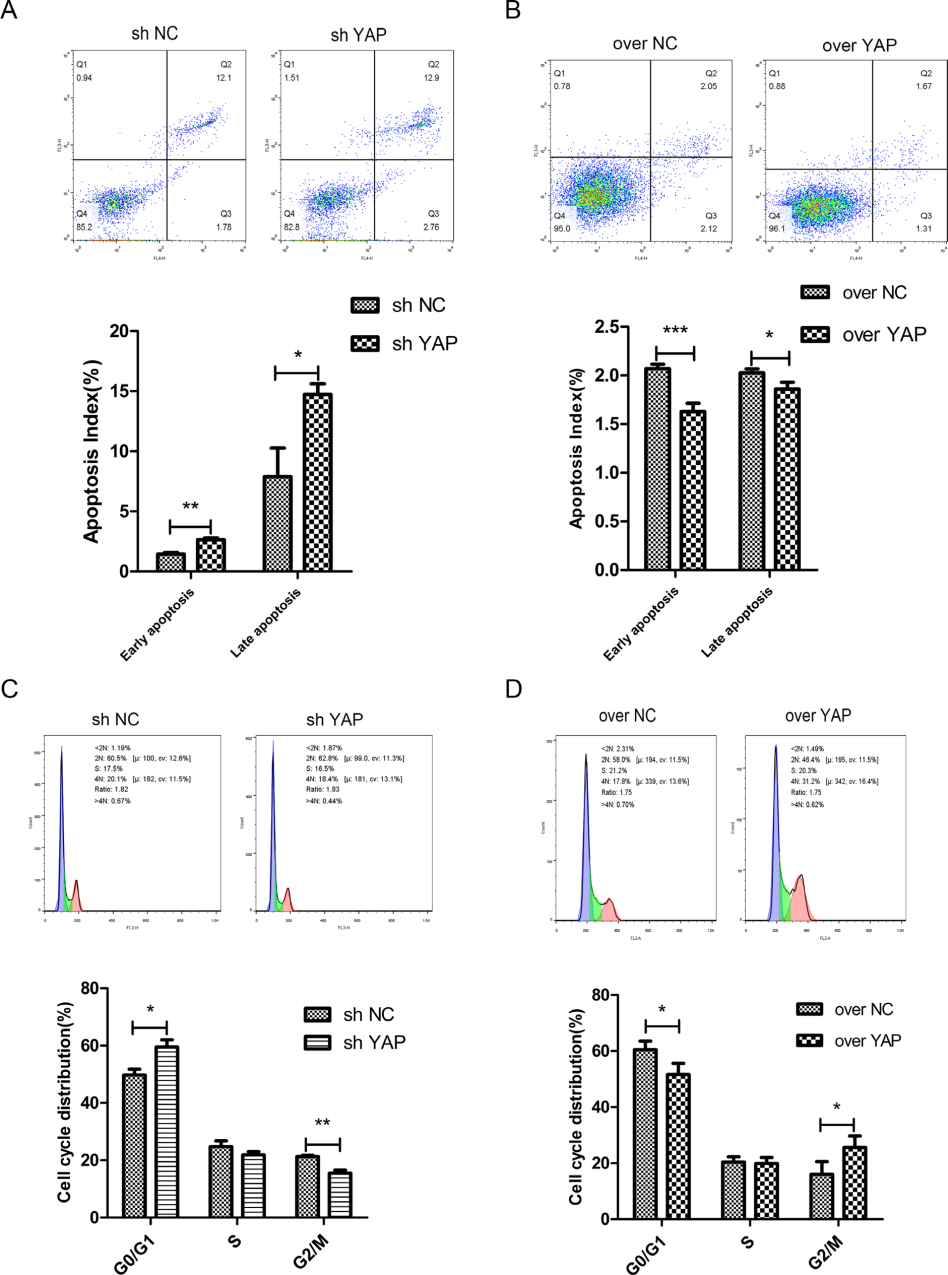
The effects of YAP knockdown or overexpression on cell apoptosis and cell cycle transition (**A**, **B**) The cell cycle phase distribution was evaluated by FACS. Shown are representative images of separate experiments. The cell cycle distribution was calculated and expressed as mean ± SD of 3 separate experiments. (**C**, **D**) CAL27 cells apoptosis were measured by Annexin-V-APC/7AAD staining. Statistical analysis of flow cytometry results. The presented columns are given as the means ± SD. ^*^p < 0.05, ^**^P < 0.01, ^***^p < 0.001.

The impact of YAP on cell apoptosis and cell cycle were investigated by flow cytometry. The results indicated that knockdown of YAP (sh YAP) led to the increased of apoptotic rates as compared to those of the control group (sh NC) (Figure 5A); in contrast, the early and late apoptotic cells in YAP over expression group (over YAP) were significantly less than those in the control group (over NC) (Figure 5B). Meanwhile, cell cycle analysis showed that knockdown of YAP led to cell cycle arrested in G0/G1 phase (Figure 5C). And overexpression of YAP (over YAP) expedited progression into the G2/M phase when compared with control group (over NC) (Figure 5D), which can partly account for the augment of proliferation.

### C-MYC and BCL-2 were functionally involved in YAP-regulated OSCC tumorigenesis

**Figure 6:**
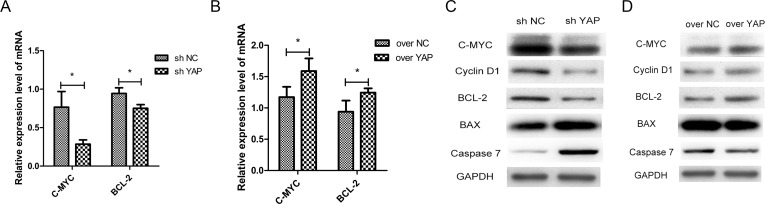
C-MYC and BCL-2 were the target genes of YAP-TEAD1 transcriptional active complex (**A**, **B**) The changes of mRNA levels of C-MYC and BCL-2 after YAP knockdown or overexpression. (**C**, **D**) Western blot analysis of the expression of C-MYC and BCL-2 and other cell cycle-related and apoptosis-related proteins, such as Cyclin D1, BAX, Caspase7. GAPDH was used as a loading control. ^*^p < 0.05.

The results of cell proliferation and apoptosis in this study indicated that YAP may promote the proliferation and inhibit cell apoptosis of OSCC cells. Prior studies have documented that C-MYC and BCL-2 pathway played a vital role in OSCC tumorigenesis [13, 14, 19]. To further understand the mechanisms of YAP in OSCC tumorigenesis, the C-MYC and BCL-2 pathways were examined in YAP knockdown or overexpression cells. We found that knockdown of YAP suppressed the mRNA expression of C-MYC and BCL-2 (Figure 6A), and overexpression of YAP promoted the gene expression (Figure 6B). YAP knockdown decreased C-MYC, Cyclin D1, BCL-2 and increased Caspase7 and BAX protein productions (Figure 6C). In contrast, YAP overexpression increased C-MYC, Cyclin D1, BCL-2 and decreased Caspase7 and BAX proteins (Figure 6D).

### Inhibition of YAP pharmacologically attenuated cell proliferation and increased cell apoptosis of OSCC cells

**Figure 7:**
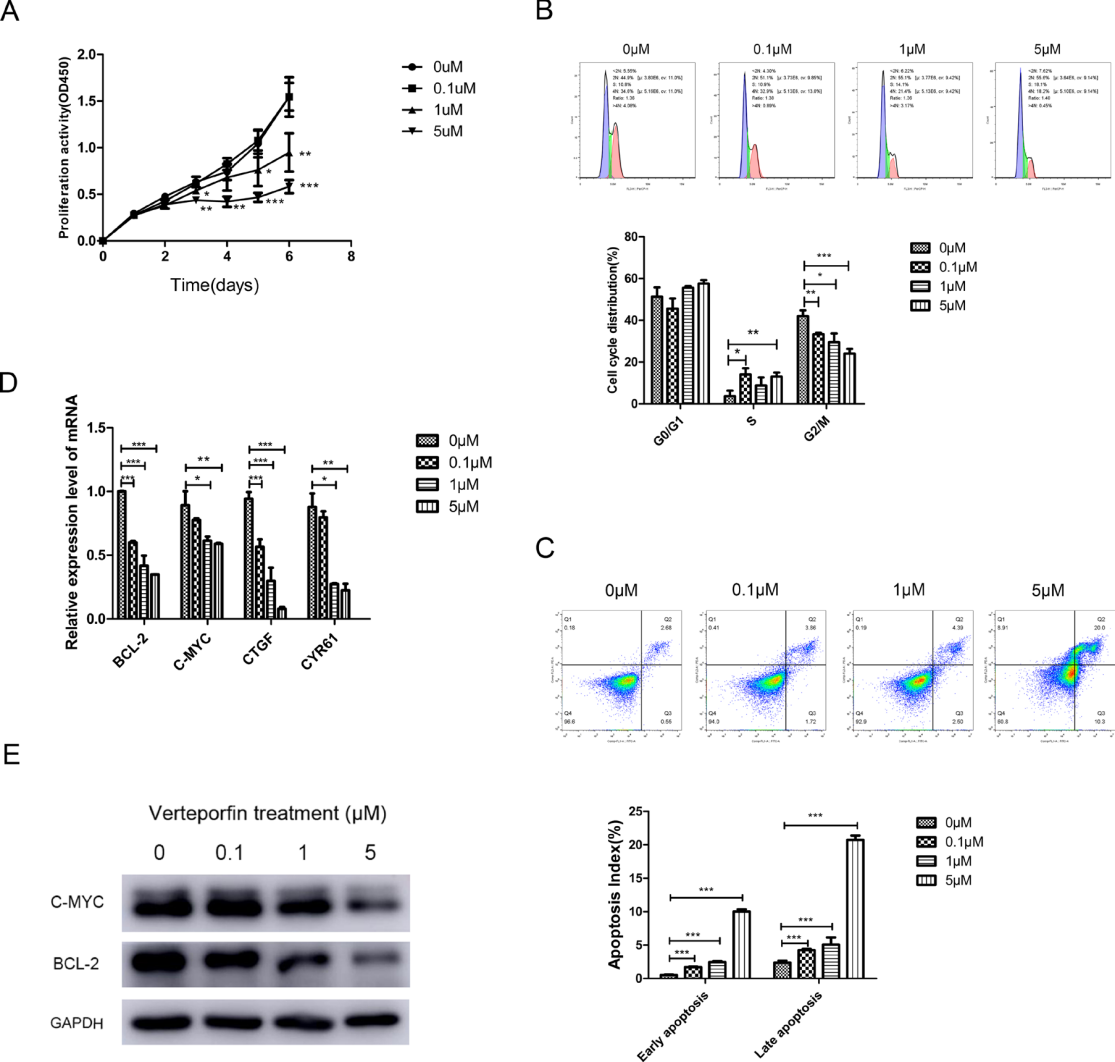
Verteporfin treatment inhibited the transcription of BCL-2 and C-MYC (**A**) Verteporfin inhibited the proliferation activity of CAL27 cell lines. (**B**) The cell cycle of CAL27 cells treated with Verteporfin arrested in S phase. (**C**) The proportion of early and late apoptosis cells increased after Verteporfin treatment. (**D**) The mRNA expression level of BCL-2 and C-MYC decreased after Verteporfin treatment, along with YAP target gene CTGF and CYR61. (**E**) The expression of BCL-2 and C-MYC proteins decreased with the increase of Verteporfin concentration. The presented columns are given as the means ± SD. ^*^p < 0.05, ^**^P < 0.01, ^***^p < 0.001.

The results of C-MYC and BCL-2 expressions indicated that BCL-2 and C-MYC might be target genes of YAP-TEAD complex. To test this, CAL27 cells were treated with different concentrations of Verteporfin, a suppressor of YAP-TEAD complex [20]. CAL27 cell proliferation activity was decreased by Verteporfin at the dose of 1 µM and 5 µM, as indicated by CCK-8 assay (Figure 7A). Meanwhile, the proportion of cells in G2/M phase decreased and the cells in early and late apoptosis increased after treatment with Verteporfin (Figure 7B–7C). Moreover, a dose-dependent reduction of BCL-2 and C-MYC was identified in both mRNA and protein levels (Figure 7D–7E).

### YAP accelerated tumorigenicity of OSCC cells *in vivo*

**Figure 8:**
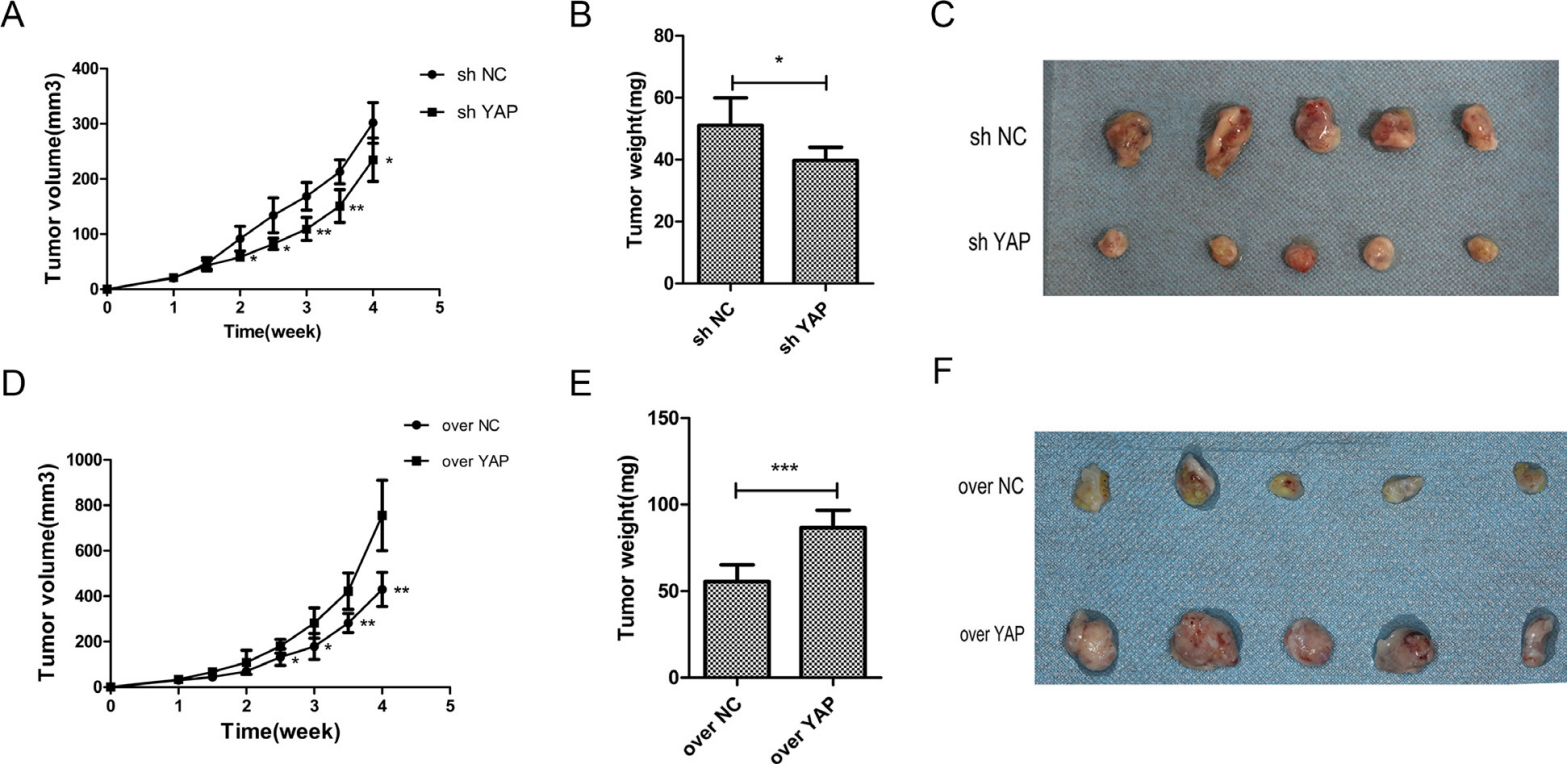
YAP accelerated tumorigenicity of OSCC cells *in vivo* (**A**, **D**) Time course analysis of tumor growth in nude mice after injection of CAL27 cells. (**B**, **E**) The tumors weight were weighed and analyzed by Student *t* test. Means ± SD were shown. (**C**, **F**) Representative images (week 4) of subcutaneous tumor xenograft in nude mice inoculated with CAL27 cells. ^*^p < 0.05, ^**^P < 0.01, ^***^p < 0.001.

The effects of YAP gene knockdown or overexpression on tumor growth were investigated in nude mice. OSCC cells with stable expression of either control vector (sh NC) or YAP knockdown (sh YAP) were embedded into the nude mice. The growth of tumors derived from YAP knockdown cells was depressed as compared to those tumors derived from control vector (sh NC). The tumor volumes and weights were reduced after YAP knockdown (Figure 8A–8C). In contrast, YAP overexpression led to the acceleration of tumor growth (Figure 8D–8F).

## DISCUSSION

It is important to investigate cancer-related genes and to identify differences in the genetics between cancer and normal cells in cancer-targeted therapy. Nowadays, a lot of efforts have been paid for the study of oral squamous cell carcinoma, but the pathogenesis and molecular mechanism of oral cancer cell apoptosis, proliferation and survival still remain unclear [21–24]. Several of studies have shown that the abnormality of YAP expression and cell proliferation, growth and tumorigenesis are positively correlated [9, 25, 26]. However, the detailed role of YAP in cancer cell proliferation and apoptosis remains controversial.

In order to explore the biological role of YAP in OSCC, the expression level of YAP at the protein level was tested by IHC staining. The results showed that the expression level of YAP in OSCC was significantly higher than that in normal tissue adjacent toOSCC. Meanwhile, YAP expression in poorly differentiated OSCC was significantly higher than that in well-differentiated OSCC tissues. To further elucidate the potential biological effects of YAP in OSCC, we evaluated the different expression levels of YAP on the proliferation potential of OSCC cells. As a result, overexpression of YAP in CAL27 cell lines promoted cell proliferation, whereas YAP silencing prohibited cell proliferation. Flow cytometry results indicated that YAP could protect the cell from apoptosis and promote cell cycle, which may contribute to tumor formation function. At the same time, the tumor xenograft model in nude mice revealed that knockdown of YAP reduced the tumorigenesis, while overexpression of YAP increased the capability of tumorigenesis of CAL27 cell lines.

Sustaining proliferative signaling and resisting cell death are two of biological capabilities that human tumors acquired [27]. The unlimited proliferation in cancer reflects of dysregulation of cell cycle or apoptosis, which leads to tumor formation. The cell cycle transition from the G1 phase to the S phase is the major regulatory checkpoint in this process [28]. Our results showed that the altering expressing levels of YAP affected the cell cycle progression. The results provided the molecular evidence which could explain the observed cell cycle changes while YAP knockdown or overexpression. Regardless of the exact function of C-MYC in regulating gene expression, studies to date support the notion that activation of C-MYC results in a genomic program that promotes ribosome biogenesis, cell growth and subsequently cell proliferation [29]. Our findings illustrated that knockdown of YAP prohibited the expression of C-MYC, while overexpression of YAP showed the opposite effects both in mRNA and protein levels, suggesting that YAP could regulate C-MYC transcriptional activity. The effect of Verteporfin is to inhibit the transcription activity of YAP target genes, and the changing trends of C-MYC confirmed our hypothesis. According to the recent reports [14, 15], YAP is a stimulator to C-MYC transcription, which is parallel to our finding. These data suggested that YAP is involved in the regulation of C-MYC in OSCC.

In addition, the BCL-2 family proteins play a key role in the regulation of cell death and are capable of modulating multiple cell death mechanisms including apoptosis, necrosis, and autophagy [30]. There are studies showing that Hippo-YAP signal pathway participates in the regulation of cell death mediated by complex signaling networks [31]. MCL-1 is a BCL-2 family gene and the transcription activity is reported to be regulated by YAP in liver [32]. After that, a study concludes that BCL-2 is identified as a direct target gene of YAP-TEAD1 active complex [19]. Our results showed that knockdown of YAP augmented cell apoptosis and the expression level of BCL-2 decreased. In contrast, overexpression of YAP showed opposite effects. On the basis of our and other’s studies, we hypothesize that YAP may maintain BCL-2 transcription activity and modulate the apoptosis of OSCC.

Before our work, there are some studies about YAP expression in OSCC. Despite the fact, it remains unclear how YAP could increase the oncogenic activity of OSCC. Turato *et al.* and Xiao *et al.* reported that YAP could exert part of its oncogenic activities by modulating the expression of C-MYC in liver cancer [14, 15]. But there are other factors involved in the indirect regulation. Although our results suggest that YAP could regulate the expression of the C-MYC expression in OSCC, it is not clear whether C-MYC is a direct or indirect target gene of YAP. At the same time, whether the C-MYC and BCL-2 signaling pathways are interacting with each other remains unknown, or that they act independently to facilitate OSCC progression. These questions are worthy to further study in OSCC.

**Figure 9:**
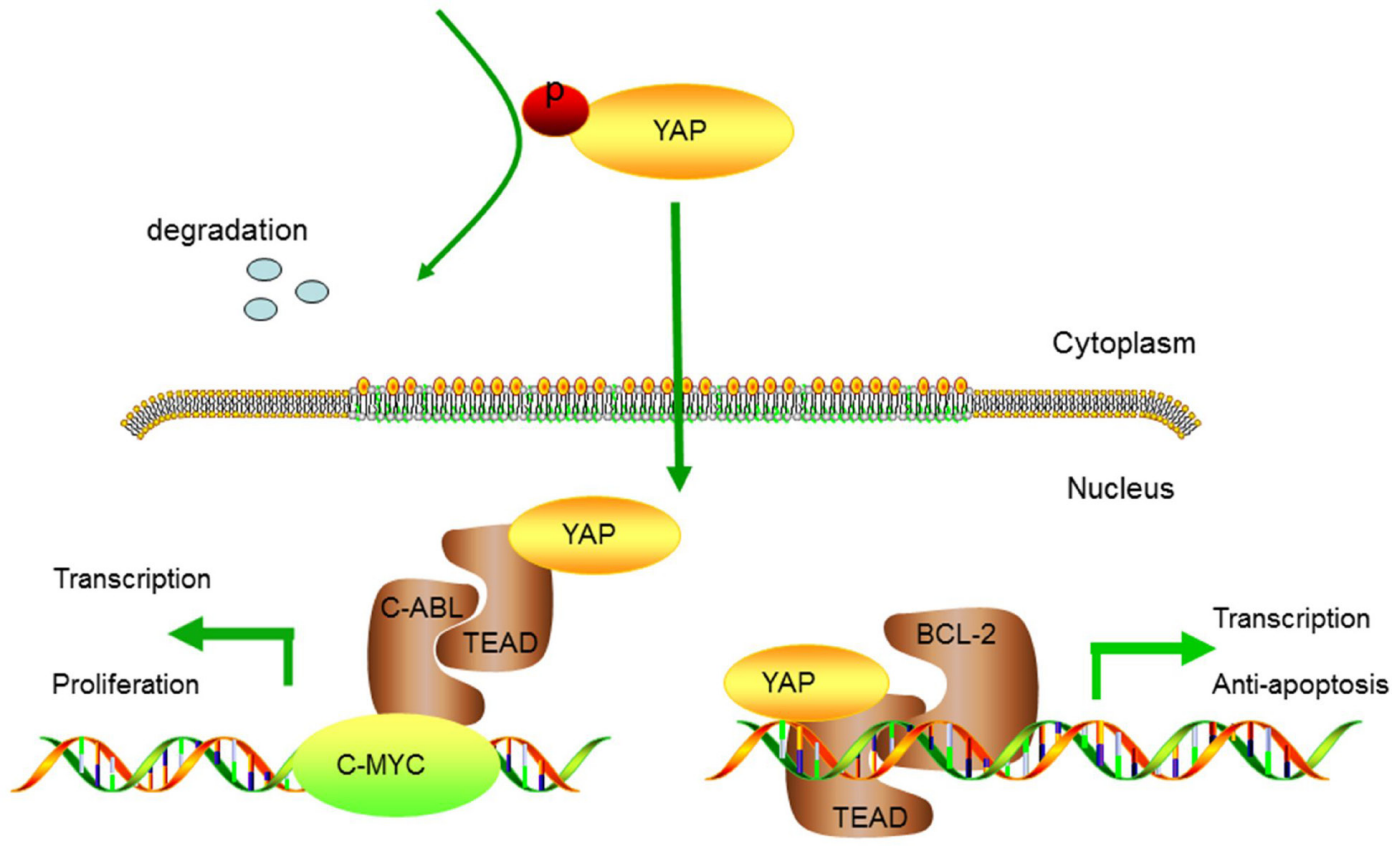
Hypothetical model of YAP-regulated tumorigenesis in OSCC Inactivated of Hippo signal pathway determines an increase of YAP and its nuclear translocation. YAP could indirectly or directly drive C-MYC transcriptional activity, which leads to sustained cell proliferation. Meanwhile, YAP is able to increase the transcription of BCL-2 gene and result in resisting cell apoptosis.

In conclusion, our study indicates that the expression of YAP is related to the pathological differentiation degree of OSCC. The novelty of this works is that YAP can regulate tumorigenicity of OSCC by modulating proliferation and apoptosis. Firstly, YAP could drive C-MYC transcription indirectly or directly which lead to subsequently cell proliferation. Meawhile, YAP is able to increase the transcription of BCL-2 gene directly and result in resisting apoptosis (Figure 9). These results indicate that YAP may be a candidate tumor activator and a potential biomarker for OSCC.

## MATERIALS AND METHODS

### Cell culture

Four OSCC cell lines were used in this study, in which CAL27, KB, and TCA8113 were purchased from the CCTCC and A253 from ATCC. The CAL27 cell line were cultured with DMEM High Glucose (Hyclone, Logan, Utah) supplemented with 10% fetal bovine serum (Gibco, NY). KB and TCA8113 cells were cultured in RPMI 1640 medium (Hyclone, Logan, Utah) supplemented with 10% FBS. A253 cells were cultured in McCoy’s 5A medium (Gibco, NY) containing 10% FBS.

### Tissue samples and immunohistochemistry (IHC)

Fresh OSCC tissues and paracancerous tissues (at least 1cm of distance from the edge of the tumor) were obtained from patients who were undergoing surgical resection of the primary tumor at the Stomatological Hospital Shandong University. A total of 15 normal tissue samples and 56 OSCC samples with definitive clinicopathological and histological diagnosis were used in the study. All human tissue samples were obtained and analyzed in accordance with procedures approved by the ethics committee of Stomatological Hospital Shandong University. IHC staining was carried out using commercially available antibodies. Endogenous peroxidase activity and nonspecific antigens (ZSGB-BIO, Beijing) were blocked using 3% H2O2 and serum. Then slides were incubate with YAP antibody (CST, Danvers, USA) at 4°C for 8 hours, and then horseradish peroxidase conjugated secondary antibody for half an hour at room temperature. Detecting signals with DAB (ZSGB-BIO, Beijing) and hematoxylin (Solarbio, Beijing) was used for nuclei counterstaining. The expression level of YAP was quantified according to the visual scoring system described by Marechal *et al* [33].

### YAP knockdown and overexpression in CAL27 cells by lentivirus particles

LV5-YAP lentivirus (over YAP) used for YAP overexpression and corresponding negative control lentivirus (over NC), as well as lentiviral constructs expressing LV3-YAP shRNA (sh YAP) and matched empty lentivirus (sh NC) were purchased from Genemart (Shanghai). The insertion sequences in the transfected cells were confirmed by DNA sequencing. The transfection effect was analyzed by fluorescence microscope after 48 hours. Transfected cells were treated with 1 μg/mL puromycin for 2 weeks. Then, subsequent experiments were performed in cells that were knockdown or overexpression of YAP.

### Quantitative RT-PCR

According to the manufacturer instructions, total RNA was extracted from the cells using the Trizol reagent (TAKARA, China). The cDNA was prepared using a PrimeScript RT reagent kit (TAKARA, China).Then complementary DNA and SYBR Green (TAKARA, China) were used for RT-PCR. Amplification conditions were as follows: 95°C for 15s, followed by 45 cycles at 60°C for 60s, and 72°C for 60s in LightCycler®480II(Roche). The results of fold-changes in mRNA levels were calculated by using a 2-ΔΔCt method [34]. Primers used for RT-PCR were listed in Table 1.

**Table 1:** The primers used for RT-PCR

Name	Primers	Conditions
YAP	F-TCTTACACCGTGCTGCCATT R-AGCACTGTGCCAGGTATCAC	95°C for 15 s, 60°C for 1 min, 72°C for 1min
CTGF	F- TCTCCAACCTC TCCTA CTAC R- GCACGTAGTC TTCGATCACT	95°C for 15 s, 60°C for 1 min, 72°C for 1min
CYR61	F-CCTTGTGGACAGCCAGTGTA R-ACTTGGGCCGGTATTTCTTC	95°C for 15 s, 60°C for 1 min, 72°C for 1min
ANKRD	F-AGTAGAGGAACTGGTCACTGG R-TGGGCTAGAAGTGTCTTCAGAT	95°C for 15 s, 60°C for 1 min, 72°C for 1min
BCL-2	F- CGACGACTTCTCCCGCCGCTACCGC R- CCGCATGCTGGGGCCGTACAGTTCC	95°C for 15 s, 60°C for 1 min, 72°C for 1min
C-MYC	F-CCCGCTTCTCTGAAAGGCTCTC R-CTCTGCTGCTGCTGCTGCTGGTAG	95°C for 15 s, 60°C for 1 min, 72°C for 1min
GAPDH	F-GCACCGTCAAGGCTGAGAAC R-TGGTGAAGACGCCAGTGGA	95°C for 15 s, 60°C for 1 min, 72°C for 1min

### Western blotting

Total proteins were collected in a lysis buffer containing both phosphatase and protease inhibitors. Protein concentrations was determined using a bicinchoniniacid assay (Solarbio, Beijing). The subsequent steps of Western blotting were following the standard protocols. Antibody against GAPDH was obtained from ProteinTech. Antibodies against YAP, BCL-2, p-YAP (Ser127), p-YAP (Ser397), Cyclin D1, Caspase7, C-MYC were purchased from Cell Signaling Technology(Danvers, MA). Histone H3 and Verteporfin were purchased from Wanleibio (Shenyang, China) and Sigma Aldrich (St Louis, USA), respectively.

### Cell viability assay

Virus-infected cells were seeded in 96-well plate at the density of 3500 cells/well. The viability of infected cells was examined by CCK-8 (DOJINDO) assay for up to 7 days. In short, 10 µl of CCK8 working solution was added to each well and incubated at 37°C for 2 hours. The absorbance was measured at the wavelengths of 450 nm.

### EdU-based proliferation assay

Cell proliferation was investigated using an EdU kit (RIBOBIO, Guangzhou) following manufacturers’ instructions. The cells were cultured with 10 μM EdU working solution in their specific culture medium for 2 hours in the dark. The cells were then fixed in 4% polyformaldehyde containing PBS for 15 minutes. After that, the cells were treated with 2 mg/ml glycine and 0.5% Triton X-100. At last, Hoechst 33342 working solution was added to each well and incubated for 30 minutes in the dark. The images were analyzed by an Olympus microscope.

### Colony formation assay

Approximately 1000 virus-infected cells/well were seeded in triplicate into 6-well plates. The medium was changed every 4 days. After 9–14 days of growth, the cells were fixed with 4% polyformaldehyde and stained with crystal violet. Visible colonies (more than 50 cells) were counted according to the cell numbers in each colony.

### Flow cytometry analysis of cell cycle

The cell cycle distribution was detected through a FACSCalibur flow cytometer (Becton Dickinson, San Jose, CA). Approximately 1 × 10^6^ cells were harvested and fixed with 75% ethanol for 4 hours. Then the cells were washed with PBS and stained with propidium iodide working solution for 30 minutes in the dark. After that, the stained cells were analyzed by flowcytometry. Cell cycle distribution was analyzed using FlowJo_V10 software. For each experiment, 20000 events per sample were recorded. The proportion of cells in G0/G1, S and G2/M phases were represented as DNA histograms.

### Flow cytometry analysis of cell apoptosis

Cells were collected, and the extent of apoptosis was detected using an Annexin-V-APC staining kit (Sungene Biotech) following the manufacturer’ instructions. Briefly, about 20000 cells were suspended in 500 μl of binding buffer. The cells were incubated with 5 μl Annexin V-APC for 10 minutes and incubated with 5 μl 7AAD solution for 5 minutes in darkness at room temperature. Cell apoptosis was then analyzed by flow cytometry.

### Xenograft tumor model

The female nude mice (BALB/c mice, 4 weeks old) used in the study were provided by the experimental animal center of Shandong University. Approximately 2 × 10^6^ cells transfected with lentivirus were injected subcutaneously on both dorsal sides of BALB/c mice. The size of tumors was measured every 3–4 days. After 30 days, animals were sacrificed and tumors were weighed.

### Ethics statement

The animal experiment was approved by the ethics committee of Stomatological Hospital Shandong University. All the animal procedures in this study were conducted in accordance with National Institutes of Health Guidelines for the Care and Use of Laboratory Animals.

### Statistical analysis

The data was analysis with SPSS software version 21 (IBM Corporation, Armonk). The results were expressed as mean ± SD. The significance of mean difference between test and control samples was compared by using Student’s *t*-test. *p* < 0.05 indicates a significant difference.

## References

[1] Warnakulasuriya S (2009). Global epidemiology of oral and oropharyngeal cancer. Oral Oncol.

[2] Chi AC, Day TA, Neville BW (2015). Oral cavity and oropharyngeal squamous cell carcinoma—an update. CA Cancer J Clin.

[3] Liu-Chittenden Y, Huang B, Shim JS, Chen Q, Lee SJ, Anders RA, Liu JO, Pan D (2012). Genetic and pharmacological disruption of the TEAD-YAP complex suppresses the oncogenic activity of YAP. Genes Dev.

[4] Harvey KF, Zhang X, Thomas DM (2013). The Hippo pathway and human cancer. Nat Rev Cancer.

[5] Guo L, Teng L (2015). YAP/TAZ for cancer therapy: opportunities and challenges (review). Int J Oncol.

[6] Bu J, Bu X, Liu B, Chen F, Chen P (2015). Increased Expression of Tissue/Salivary Transgelin mRNA Predicts Poor Prognosis in Patients with Oral Squamous Cell Carcinoma (OSCC). Med Sci Monit.

[7] Muramatsu T, Imoto I, Matsui T, Kozaki K, Haruki S, Sudol M, Shimada Y, Tsuda H, Kawano T, Inazawa J (2011). YAP is a candidate oncogene for esophageal squamous cell carcinoma. Carcinogenesis.

[8] Chan LH, Wang W, Yeung W, Deng Y, Yuan P, Mak KK (2014). Hedgehog signaling induces osteosarcoma development through Yap1 and H19 overexpression. Oncogene.

[9] Ling HH, Kuo CC, Lin BX, Huang YH, Lin CW (2017). Elevation of YAP promotes the epithelial-mesenchymal transition and tumor aggressiveness in colorectal cancer. Exp Cell Res.

[10] Fernandez A, Squatrito M, Northcott P, Awan A, Holland EC, Taylor MD, Nahle Z, Kenney AM (2012). Oncogenic YAP promotes radioresistance and genomic instability in medulloblastoma through IGF2-mediated Akt activation. Oncogene.

[11] Ren K, Li T, Zhang W, Ren J, Li Z, Wu G (2016). miR-199a-3p inhibits cell proliferation and induces apoptosis by targeting YAP1, suppressing Jagged1-Notch signaling in human hepatocellular carcinoma. J Biomed Sci.

[12] Chen YJ, Lin SC, Kao T, Chang CS, Hong PS, Shieh TM, Chang KW (2004). Genome-wide profiling of oral squamous cell carcinoma. J Pathol.

[13] Kaur M, Cole MD (2013). MYC acts via the PTEN tumor suppressor to elicit autoregulation and genome-wide gene repression by activation of the Ezh2 methyltransferase. Cancer Res.

[14] Xiao W, Wang J, Ou C, Zhang Y, Ma L, Weng W, Pan Q, Sun F (2013). Mutual interaction between YAP and c-Myc is critical for carcinogenesis in liver cancer. Biochem Biophys Res Commun.

[15] Turato C, Cannito S, Simonato D, Villano G, Morello E, Terrin L, Quarta S, Biasiolo A, Ruvoletto M, Martini A, Fasolato S, Zanus G, Cillo U (2015). SerpinB3 and Yap Interplay Increases Myc Oncogenic Activity. Sci Rep.

[16] Su Z, Yang Z, Xu Y, Chen Y, Yu Q (2015). Apoptosis, autophagy, necroptosis, and cancer metastasis. Mol Cancer.

[17] Adams JM, Cory S (2007). The Bcl-2 apoptotic switch in cancer development and therapy. Oncogene.

[18] Duan Y, He Q, Yue K, Si H, Wang J, Zhou X, Wang X (2017). Hypoxia induced Bcl-2/Twist1 complex promotes tumor cell invasion in oral squamous cell carcinoma. Oncotarget.

[19] Song Y, Fu J, Zhou M, Xiao L, Feng X, Chen H, Huang W (2016). Activated Hippo/Yes-Associated Protein Pathway Promotes Cell Proliferation and Anti-apoptosis in Endometrial Stromal Cells of Endometriosis. J Clin Endocrinol Metab.

[20] Feng J, Gou J, Jia J, Yi T, Cui T, Li Z (2016). Verteporfin, a suppressor of YAP-TEAD complex, presents promising antitumor properties on ovarian cancer. Onco Targets Ther.

[21] Lu M, Chen WH, Wang CY, Mao CQ, Wang J (2017). Reciprocal regulation of miR-1254 and c-Myc in oral squamous cell carcinoma suppresses EMT-mediated metastasis and tumor-initiating properties through MAPK signaling. Biochem Biophys Res Commun.

[22] Fujii M, Katase N, Lefeuvre M, Gunduz M, Buery RR, Tamamura R, Tsujigiwa H, Nagatsuka H (2011). Dickkopf (Dkk)-3 and beta-catenin expressions increased in the transition from normal oral mucosal to oral squamous cell carcinoma. J Mol Histol.

[23] Ahn MY, Yoon JH (2017). Histone deacetylase 8 as a novel therapeutic target in oral squamous cell carcinoma. Oncol Rep.

[24] Pan H, Gu LQ, Liu BJ, Li YP, Wang YH, Bai XN, Li L, Wang BS, Peng Q, Yao Z, Tang ZG (2017). Tropomyosin-1 acts as a potential tumor suppressor in human oral squamous cell carcinoma. Plos One.

[25] Wang JY, Ma LF, Weng WH, Qiao YX, Zhang Y, He JT, Wang HM, Xiao WF, Li LL, Chu QH, Pan QH, Yu YC, Sun FY (2013). Mutual Interaction Between YAP and CREB Promotes Tumorigenesis in Liver Cancer. Hepatology.

[26] You B, Yang YL, Xu ZD, Dai YY, Liu S, Mao JH, Tetsu O, Li H, Jablons DM, You L (2015). Inhibition of ERK1/2 down-regulates the Hippo/YAP signaling pathway in human NSCLC cells. Oncotarget.

[27] Hanahan D, Weinberg RA (2011). Hallmarks of Cancer: The Next Generation. Cell.

[28] Zhao FJ, Lin TX, He W, Han JL, Zhu DJ, Hu KS, Li WC, Zheng ZS, Huang J, Xie WL (2015). Knockdown of a novel lincRNA AATBC suppresses proliferation and induces apoptosis in bladder cancer. Oncotarget.

[29] Stine ZE, Walton ZE, Altman BJ, Hsieh AL, Dang CV (2015). MYC, Metabolism, and Cancer. Cancer Discov.

[30] Yip KW, Reed JC (2008). Bcl-2 family proteins and cancer. Oncogene.

[31] Fallahi E, O’Driscoll NA, Matallanas D (2016). The MST/Hippo Pathway and Cell Death: A Non-Canonical Affair. Genes.

[32] Dong JX, Feldmann G, Huang JB, Wu S, Zhang NL, Comerford SA, Gayyed MF, Anders RA, Maitra A, Pan DJ (2007). Elucidation of a universal size-control mechanism in Drosophila and mammals. Cell.

[33] Marechal R, Demetter P, Nagy N, Berton A, Decaestecker C, Polus M, Closset J, Deviere J, Salmon I, Van Laethem JL (2009). High expression of CXCR4 may predict poor survival in resected pancreatic adenocarcinoma. Br J Cancer.

[34] Livak KJ, Schmittgen TD (2001). Analysis of relative gene expression data using real-time quantitative PCR and the 2(T)(-Delta Delta C) method. Methods.

